# C2M-Mamba: drug-drug interaction prediction based on cross-modal cross-Mamba

**DOI:** 10.1186/s12859-026-06420-4

**Published:** 2026-03-11

**Authors:** Shanwen Zhang, Chuanlei Zhang, Dengwu Wang

**Affiliations:** 1 College of Electronic Information, Xijing University, Xi’an, 710123 China; 2 School of Artificial Intelligence, Tianjin University of Science and Technology, Tianjin, 300222 China

**Keywords:** Drug-drug interaction prediction (DDIP), Cross-modal learning, Cross-Mamba (CroMamba), Cross-modal CroMamba model (C2M-Mamba)

## Abstract

Accurately predicting potential drug-drug interactions (DDIs) from multimodal data is critical for medication safety and adverse drug reaction prevention. Existing methods face challenges in modeling long-range dependencies and effectively integrating heterogeneous features from structured molecular data and unstructured text. To address these limitations, we propose C2M-Mamba, a cross-modal framework that integrates convolutional neural networks, Mamba, and cross-Mamba (CroMamba) to capture discriminative features from drug descriptions, SMILES sequences, and social media texts. The model efficiently handles long-range dependencies through state space models while enabling effective cross-modal fusion. Comprehensive evaluations on the DDIExtraction2013 dataset demonstrate that C2M-Mamba outperforms 10 state-of-the-art baselines, achieving 82.37% precision, 80.98% F1-score, and 88.73% AUC. The proposed approach also exhibits robust performance in handling class imbalance and provides interpretable predictions, offering a reliable solution for multimodal DDI prediction with potential applications in pharmacovigilance and personalized medicine.

## Introduction

Complex diseases, such as cancer, cardiovascular disorders and diabetes, are usually treated through multi-drug combination therapy, which can enhance the therapeutic effect and prevent drug resistance, but it also increases the risk of adverse drug reactions (ADRs) due to unforeseen adverse DDIs, where DDI refers to the significant impact of one drug on the pharmacodynamics or pharmacokinetics of another when two or more drugs are used simultaneously or sequentially, and ADR as adverse DDI, usually results in severe clinical consequences, including unexpected toxicity, reduced treatment effectiveness, patient hospitalization, and even fatal, while many DDIs remain unknown [1]. Among the over 8 million possible drug pairs, the known DDIs in the existing datasets account for less than 10%, leaving a large number of potential DDIs that have not yet been explored. Although traditional DDI prediction (DDIP) methods through laboratory experiments and clinical trials are reliable, they are costly, time-consuming, have low prediction results, and it is unrealistic to detect all possible drug combinations through experimental methods with the number of drugs increase. Therefore, it is important to develop advanced DDIP methods. Many DDIP methods based on hybrid deep learning (DL) models have been presented to meet this demand by leveraging structured molecular data and unstructured social media text, and the most existing DL models independently extract DDI features, but ignore their interactions during the feature learning process, resulting in low-accuracy [2]. Feature fusion is usually implemented through simple concatenation followed by fully connected layers, which ignores the inherent heterogeneity among drugs. The cross-attention mechanism can effectively integrate multimodal features, but its high complexity and sensitivity to irrelevant binding features may lead to unstable interactions [3]. Existing DL-based DDIP methods widely utilize multimodal characteristics of drugs, such as chemical structures (like SMILES sequences, molecular diagrams), biological properties (such as targets, enzymes), and clinical phenotypes (such as side effects, indications) [4]. As a powerful and universal model for processing sequential data, Transformer has performed exceptionally well in various types of tasks. However, it is limited due to its high quadratic computational complexity [5]. Recently, State Space Models (SSMs) such as Mamba have emerged as a promising alternative to DDIP, aiming to address the limitations of the traditional DL models and Transformer with a linear complexity of O(*n*) [6].

Most of the existing DDIP methods usually concatenate different features or use basic attention mechanisms, making it difficult to achieve deep integration among DDIP-related features. Transformer has powerful performance, but when dealing with long SMILES sequences and long texts, its computational complexity grows exponentially, which limits its practical application effect.

Motivated by three fundamental limitations in current multimodal fusion approaches: the computational burden of full cross-attention (O(N^2^)), the simplistic nature of concatenation-based fusion, and inadequate long-range dependency modeling in existing architectures, a cross-modal CroMamba model (C2M-Mamba) is constructed to capture long-range dependencies while preserving the local feature extraction, and enhance DDIP performance by integrating multimodal features. The main contributions of this paper are given as follows:

1. State space model (SSM) is used to effectively capture the multimodal features from molecular structure data (SMILES) and social media text.
2. A cross-modal attention fusion module is designed to integrate the multimodal features.
3. Extensive experiments on the public DDI dataset demonstrate that C2M-Mamba outperforms 4 state-of-the-art baselines.

The rest of the paper is organized as follows. Sect. “Related work” reviews related work. Sect. “Cross-modal CroMamba model (C2M-Mamba)” describes the proposed model C2M-Mamba in detail. Sect. “Experiments” presents extensive experimental comparisons with 6 state-of-the-art methods. Sect. “Conclusion” concludes the paper and discusses potential future research directions.

## Related work

With the increasing popularity of multi-drug combination therapy in clinical practice, DDIP has become one of the core challenges in ensuring medication safety. Driven by DL and bigdata processing technologies, a large number of DDIP studies have been presented in recent years, which can be classified into three categories: DL-based methods, Transformer-based methods, and Mamba-based methods [7, 8].

### DL-based methods

DL is an effective and reliable alternative for DDIP by leveraging complex patterns from multimodal data. Zhang et al. [7] reviewed the recent DL methods applied to DDIP from biomedical literature, briefly described each method, systematically compared their performance in the DDI corpus, summarized the advantages and disadvantages of these DL models for this task, and discussed some challenges and future prospects of extracting DDI through DL models. This review provides useful guidance for interested researchers to further advance DDIP from literature by bioinformatics algorithms. Several recent studies have significantly advanced DDI prediction through innovative architectures. Molormer introduces a lightweight self-attention mechanism that explicitly captures spatial structures in molecular graphs [8], while AMDE employs multidimensional attention to encode features from both SMILES sequences and molecular graphs [9], and MDDI-SCL explores supervised contrastive learning for multi-type interaction prediction [10]. Jung et al. [11] utilized biomedical literature to propose a hierarchical attention DL model based DDIP, and used sentence and sequence embedding methods to extract the representation vectors that effectively capture drug attributes. Li et al. [12] proposed a dual-view drug representation learning network DSN-DDI for DDIP. It iteratively adopts local and global representation learning modules to simultaneously learn drug substructures from both single drugs and drug pairs. Niu et al. [13] proposed a DDIP model SRR-DDI based on substructure fine-grained representation learning of self-attention mechanism. It can enhance the robustness of substructure features and improve the performance of DDIP. Later, they proposed a dual-view DDIP model namely DAS-DDI [14], where an attention mechanism is employed to dynamically fuse multi-drug embeddings from different views, enhancing the model discrimination ability.

From the above analysis, it is found that the above DL models are difficult to effectively capture the long-range dependencies in drug sequences, especially in multi-modal DDIP in real scenarios.

### Transformer-based methods

Transformer fundamentally resolves the problem of long-range dependence through its Multi-Head Self-Attention (MHSA) mechanism. In recent years, Transformer has been introduced into the DDIP task [15]. Lin et al. [16] proposed a DDIP method namely MDF-SA-DDI based on multi-source drug fusion, multi-source feature fusion and Transformer. It is mainly composed of multi-source drug fusion and multi-source feature fusion. Yu et al. [17] constructed a DDIP method based on multimodal feature fusion by integrating BiGRU into Transformer, where BiGRU is used to extract the local features while MHSA is used to capture global features. Zhang et al. [18] constructed a Semantic Cross-Attention Transformer model for DDIP. In the model, BioBERT, Doc2Vec and graph convolutional network are utilized to embed the multimodal biomedical data into vector representation, BiGRU and Cross-Attention are adopted to capture contextual dependencies and integrate the extracted features and explicitly model dependencies between them. Zhao et al. [19] proposed a dual-source Transformer framework to effectively combine knowledge graph information with protein sequence features for DDIP. The model consists of a Dual-Source Attention Mechanism for feature extraction, a Bimodal Interaction Enhancement module for feature fusion, and a Sequential Transformer Refinement for comprehensive interaction pattern learning.

From the above analysis, it is found that the above Transformer models are limited by their high computation complexity.

### Mamba-based method

Mamba is a novel DL architecture designed to enhance the efficiency of Transformer models in handling long contexts. By utilizing SSM, it can efficiently capture long-distance dependencies with low computational complexity. Unlike traditional MHSA, Mamba uses fixed-size hidden states as memory units, enabling its computational cost to remain stable even when the input text is long [20]. Wu et al. [6] introduced a drug-target affinity prediction architecture namely MambaDTA based on SSM. The model utilizes SSM to model the drug molecules and target molecules and extract more discriminative spatial structural features efficiently and stably. Xu et al. [21] proposed a two-stage model SMILES-Mamba that leverages both unlabeled and labeled data by combining self-supervised pre-training and fine-tuning strategies. The model is pre-trained on a large corpus of unlabeled SMILES strings to capture potential chemical structures and relationships, and fine-tuned on a smaller labeled dataset specific to the task. Xie et al. [22] designed an end-to-end cross-modal fusion framework CrossMamba, which can dynamically and selectively integrate the chemical structure information of drugs with the contextual information of biomedical texts, enhancing the representation learning ability of the model.

SMILES standardizes the representation of molecular structures in string form, providing a digital basis for DDIP. By encoding atomic, chemical bond and functional group information, SMILES can be directly input into DL models (such as LSTM, Transformer), and converted into molecular embedding vectors, capturing key substructure features and calculating the similarity between drugs. Based on Mamba, MambaDTA, CrossMamba, and the above multimodal DDIP methods, a cross-modal CroMamba model (C2M-Mamba) for DDIP is constructed, and is verified on the public DrugBank dataset.

## Cross-modal CroMamba model (C2M-Mamba)

**Fig. 1:**
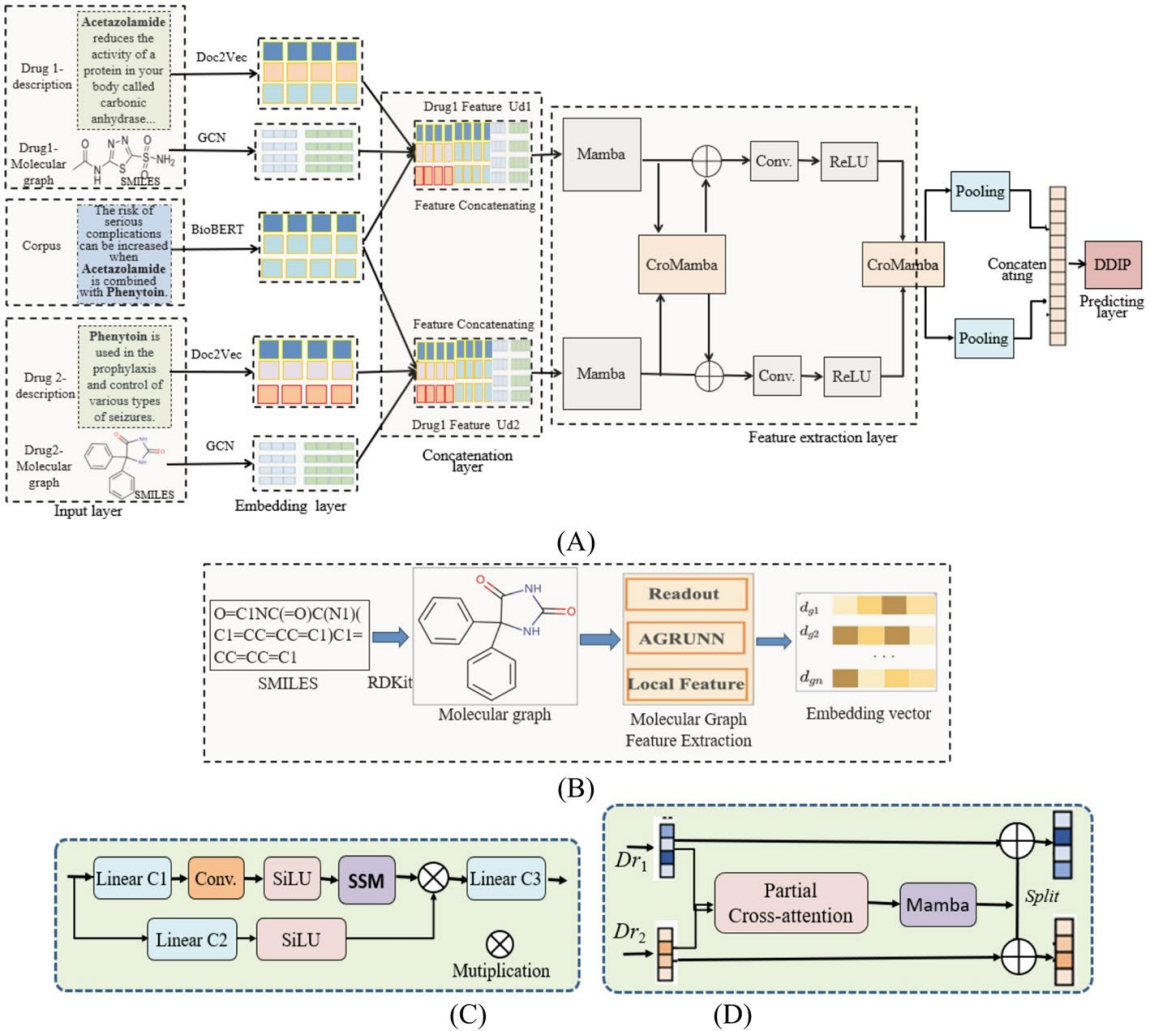
C2M-Mamba. **A** Overall framework. **B** The SMILES embedding process by SMILES2Vec to convert the SMILES strings of drug molecules into vector representations. **C** Mamba captures long-range dependencies through the Selective SSM. **D** CroMamba integrates partial cross-attention and Mamba for progressive interaction between drugs and targets

**Fig. 2:**
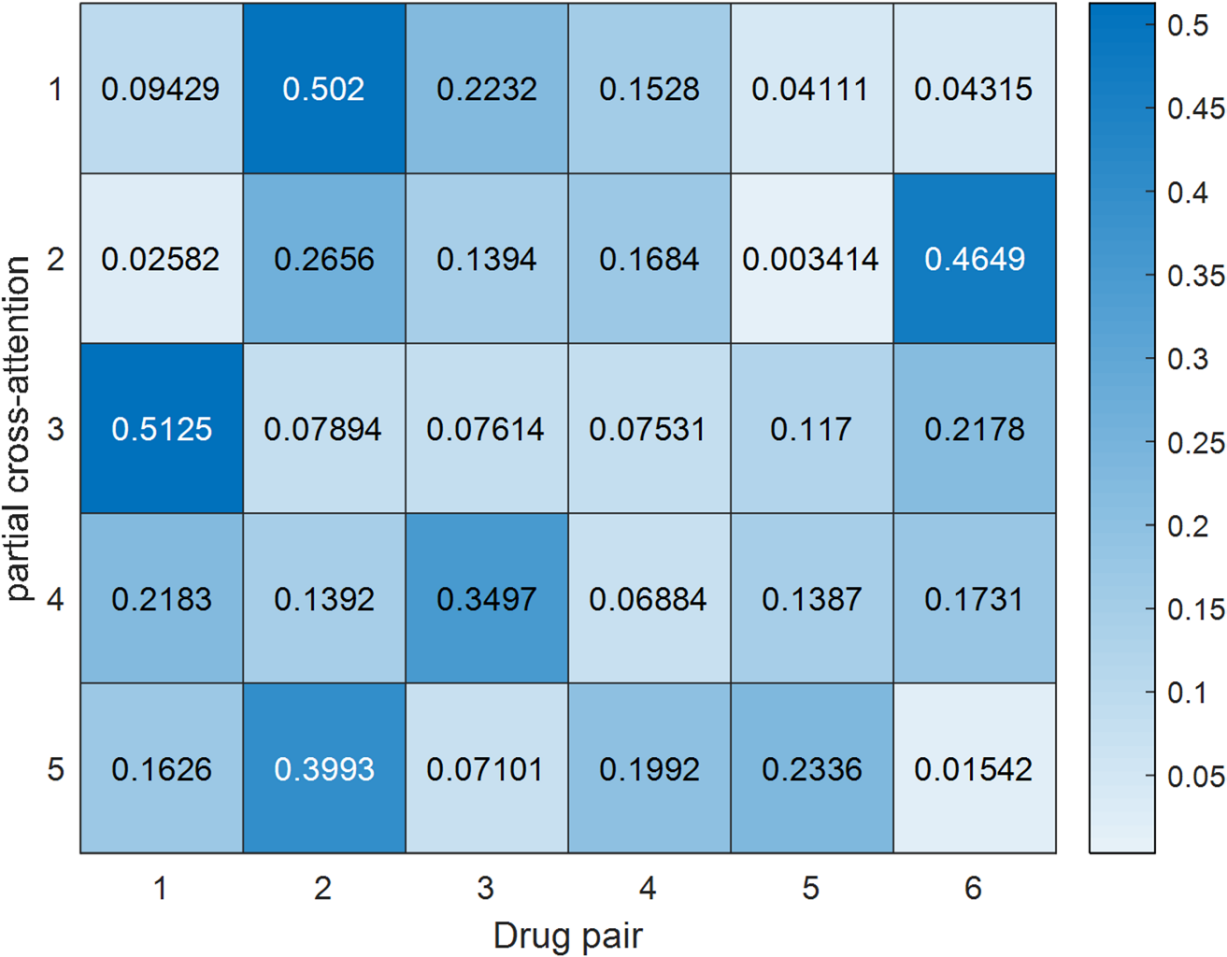
Partial cross-attention weights var interaction patterns

The overall architecture of C2M-Mamba is shown in Fig. 1, consisting of input layer, embedding layer, concatenating layer, feature extraction layer and predicting layer, where Mamba and CroMamba are two main modules. The model has two independent Mamba components to extract and integrate the multimodal features from multimodal data, such as SMILES sequences and description of drugs and social media texts. In C2M-Mamba, cross-modal refers to processing cross-modal data, CroMamba conducts cross-modal interaction and integration, and Mamba as the feature extractor for each module is used to efficiently capture the long-range dependencies within the modules. Figures 2B and C show the structures of the Mamba and CroMamba, respectively. Figure 2D shows the embedding process of drug-SMILES.

Suppose the training set $S = \left\{ {s_{1} ,s_{2} , \ldots ,s_{m} } \right\}$ containing *m* sentences and corresponding DDI label set $L = \left\{ {l_{1} ,l_{2} , \ldots ,l_{m} } \right\}$, each sentence has *n* words $s_{i} = \left\{ {w_{1} ,w_{2} , \ldots ,w_{n} } \right\}$, where drug1 and drug2 are two target drugs, and the drug-description sentences of drugA and drugB are noted as $q_{1i}$ and $q_{2i}$, respectively. The main steps of C2M-Mamba based DDIP are described as follows.

### Overall architecture

C2M-Mamba is composed of multiple components to address the fundamental challenges in multimodal DDIP. Each component plays a complementary role in processing heterogeneous drug data and capturing different aspects of drug-drug interactions.

It employs multiple embedding strategies (Word2Vec, GloVe, Graph2Vec, SMILES2Vec) to capture complementary semantic, syntactic, and structural information from different data representations, as different interaction mechanisms may be more pronounced in some representations than in others. Convolutional components, as local feature detectors, identify key chemical substructures and meaningful text phrases as interaction indicators. The Mamba layer provides effective remote dependency modeling with linear complexity, which is crucial for capturing extended context relationships and drug descriptions in long SMILES strings, where important interaction clues may be distant.

The core CroMamba module supports progressive and selective cross-modal fusion, addressing the fundamental challenge of intentionally integrating heterogeneous features while maintaining computational efficiency. Finally, the feature space is compressed through the PCA dimensionality reduction and intelligent pooling mechanism, while retaining the discriminant information to prevent overfitting and highlighting the most significant features for the final prediction.

### Vector embedding

Embedding and dimensionality reduction are two essential steps in natural language processing (NLP). Word2Vec is a widely used word embedding approach, frequently applied across various NLP tasks. Given the *i*th sentence *s*_i_ and its target drugs drug1 and drug2 in the sentence, the embedding vector *Ew* of *s*_i_ is obtained by Word2Vec, two drug-description vectors Eqs. 1 and 2 of two target drugs are calculated by GloVe tool (https://nlp.stanford.edu/projects/glove/).

Graph2Vec is an algorithm based on the subgraph of the graph embedding, it is a Python library developed by Benedek Rozemberczki. The substructure in Graph2vec updates its neighborhood information through the substructure of the adjacent nodes. Graph2vec is used to convert each molecule topology graph of drug1 and drug2 into a fixed-length vector $Eg_{1}$ and $Eg_{2}$, respectively.

Based on the SMIELS string of the drug, search for the ID of each character in the vocabulary to generate the ID list of the drug, and generate the mask list of the drug based on the ID list of the drug. The ID list and the mask list of the drug are jointly used as the one-dimensional SMIELS sequence of the drug. Among them, the vocabulary list refers to a pre-constructed table with a single character as the token, and each token: is equally divided and assigned a unique ID. SMILES2Vec converts the molecular structure of drugs (represented by SMILES strings) into a vector space representation[20], as shown in Fig. 2B. The drug molecule structure is represented by SMILES string from DrugBank dataset, $Sx = [sx_{1} ,sx_{2} ,...,sx_{m} ]$, where $sx_{i}$ is the mark of the SMILES string, and *m* is the length of the string. During the preprocessing stage, sequences longer than 250 characters are filtered out. Finally, the sum of the low-dimensional embedding vectors of drug 1 and drug 2 is obtained. After concatenating these vectors, a joint representation is formed. Two embedding vectors $Es_{1}$ and $Es_{2}$ of drug1 and drug2 are obtained to represent the molecule structure SMILES, respectively.

From the above calculations, the embedded vectors of two drugs are concatenated as follows,

1 $$ \begin{gathered} E_{d1} = [Ew;Eq_{1} ;Eg_{1} ;Es_{1} ] \hfill \\ E_{d2} = [Ew;Eq_{2} ;Eg_{2} ;Es_{2} ] \hfill \\ \end{gathered} $$

### Feature extraction

#### Feature extraction by Mamba

The embedding vector of the drug is subjected to feature extraction by Mamba to obtain the feature vector of the drug, as shown in Fig. 2C, calculated as follows:

2 $$ \begin{gathered} Dr_{1} = Mamba(E_{d1} ) \hfill \\ Dr_{2} = Mamba(E_{d2} ) \hfill \\ \end{gathered} $$

where $Dr_{1} ,Dr_{2}$ represent the feature vectors of the two drugs respectively, and *Mamba*(.) is the feature extraction operation of the drug embedding vector.

#### Feature fusion by CroMamba

CroMamba is designed to implement progressive feature fusion by partial cross-attention with Mamba, as shown in Fig. 2D. It integrates three key innovations: partial cross-attention that selectively focuses on the most relevant cross-modal features, reducing complexity to O(N), Mamba-based state space models for efficient long-range dependency modeling, and progressive fusion that enables local-to-global feature integration.

It is used to capture the relationships between adjacent features, and in the deep integration stage, the SSM of Mamba is utilized to model global long-range dependencies, achieving stable feature fusion from local to global. Specifically, in local cross-attention, the last dimension of the input feature is divided into two parts: one part participates in cross-attention calculation to extract local interaction information, and the other part remains unchanged to retain the original feature. The process of CroMamba is described as follow,

3 $$ \begin{gathered} Er_{1} = Mamba(PCroatten(Dr_{1} )) \hfill \\ Er_{2} = Mamba(PCroatten(Dr_{2} )) \hfill \\ Er_{1}{\prime} = Er_{1} \oplus Dr_{1} \hfill \\ Er_{2}{\prime} = Er_{2} \oplus Dr_{2} \hfill \\ \end{gathered} $$

where $PCroatten(.)$ is the partial cross-attention module.

$PCroatten(.)$ in Eq. (3) is used to find the most important features for DDIP within a drug pair that could be linked to DDIs. Its process is described as follows. $Dr_{1} ,Dr_{2}$ are divided the characteristics into two parts:

4 $$ \begin{gathered} Dr_{1} = [Dr_{1}^{1} ,Dr_{1}^{2} ] \hfill \\ Dr_{2} = [Dr_{2}^{1} ,Dr_{2}^{2} ]] \hfill \\ \end{gathered} $$

where $Dr_{1}^{1} ,Dr_{2}^{1}$ serve as the input for cross-attention, while $Dr_{1}^{2} ,Dr_{2}^{2}$ part remain unchanged.

$Dr_{1}^{1} ,Dr_{2}^{1}$ are processed by attention mechanism,

5 $$ \begin{gathered} CrossAttn(Dr_{1}^{1} ,Dr_{2}^{1} ) \hfill \\ = Softmax((Dr_{1}^{1} W_{Q} )(Dr_{2}^{1} W_{K} )^{T} /\sqrt {dk} )(Dr_{2}^{1} W_{V} ) \hfill \\ \end{gathered} $$

where $W_{Q} ,W_{K} ,W_{V}$ are three learnable wight matrices.

From Eq. (5), two interaction-enhanced features are generated as $Z_{1}^{1} ,Z_{2}^{1}$. Concatenate $Z_{1}^{1} ,Z_{2}^{1}$ with the retained features $Dr_{1}^{2} ,Dr_{2}^{2}$ as,

6 $$ \begin{gathered} F_{1}^{{}} = [Z_{1}^{1} ,Dr_{1}^{2} ] \hfill \\ F_{2}^{{}} = [Z_{2}^{1} ,Dr_{2}^{2} ] \hfill \\ \end{gathered} $$

Partial cross-attention can reduce the feature dimensions involved in the attention calculation, improve the computational efficiency, and retain the integrity of the original features, and is suitable for the modeling requirements of local structural sensitivity in DDIs.

The feature vector *V* of DDIP is calculated as follows,

7 $$ \begin{gathered} EF_{1}^{{}} = PCroatten({\mathrm{Re}} {\mathrm{LU(}}Conv(F_{1}^{{}} ))) \hfill \\ EF_{2}^{{}} = PCroatten({\mathrm{Re}} {\mathrm{LU(}}Conv(F_{2}^{{}} ))) \hfill \\ V = Pooling(EF_{1}^{{}} ) \oplus Pooling(EF_{2}^{{}} ) \hfill \\ \end{gathered} $$

where $Pooling(.)$ is pooling operation, $\oplus$ is concatenating operation or element-addition, and *V* is the output feature of the drug-drug pair.

Table 1 systematically compares CroMamba against conventional fusion strategies, highlighting its superior balance between computational efficiency and representational capacity.

**Table 1:** Comparative analysis of cross-modal fusion strategies

Fusion method	Computational complexity	Cross-modal alignment	Long-range modeling	Parameters
Full Cross-Attention	O(N^2^)	Excellent	Good	High
Concatenation + FC	O(N)	Poor	Limited	Low
Early Fusion + Transformer	O(N^2^)	Moderate	Good	High
CroMamba	O(N)	Excellent	Excellent	Moderate

As shown in Table 1, CroMamba achieves an optimal balance between efficiency and representation, with its linear complexity and strong capabilities in cross-modal alignment and long-sequence modeling making it particularly suitable for multimodal DDIP.

### DDIP and model training

The DDIP probability is obtained through the Sigmoid function, calculated as,

8 $$ \begin{gathered} FC = W \cdot V^{\prime} + b \hfill \\ P = Sigmoid(FC) \hfill \\ \end{gathered} $$

where *W* is the weight matrix, *b* is the bias parameter, $V^{\prime}$ is the flattened representation of the output feature of the drug-drug pair, *Sigmoid*(.) is Sigmoid function operation, *FC* is the output of the fully connected layer, and *P* represents the final prediction probability.

Similar to MDF-SA-DDI [5] and Mamba-DTA [6], C2M-Mamba employs forward propagation to compute model loss and backward propagation to iteratively update network weights via gradient descent. The learning rate is dynamically influenced by the loss value. To mitigate the issue of decreasing learning rate during optimization, cross-entropy is adopted as the loss function. The training objective of C2M-Mamba is to minimize the discrepancy between predictions and ground-truth labels, defined as:

9 $$ Loss = - \frac{1}{m}\sum\limits_{i = 1}^{m} {(l_{AB} \log \widehat{l}_{AB} + (1 - l_{AB} )\log (1 - \widehat{l}_{AB} ))} $$

where $$l_{AB}$$ and $$\overset{\lower0.5em\hbox{$\smash{\scriptscriptstyle\frown}$}}{l}_{AB}$$ by Eq. (10) are the real DDI-label for the drug pair (drugA, drugB) and predicting DDI probability of the *i*-th DDI type belonging to {Advice, Mechanism, Effect, Int, Negative}.

## Experiments

To validate the C2M-Mamba based DDIP method, a lot of experiments are conducted on the DDIExtraction2013 corpus, and compared with 9 state-of-the-art DDIP methods, i.e., Deep Attention Neural Networks (DANN) [3], Molormer [8], DSN-DDI [12], SRR-DDI [13], DAS-DDI [14], MDF-SA-DDI [16], SCATrans [18], CroMamba-DTA [22] and SmileGNN [23]. They are simply introduced as follows.DANN is a deep attention neural networks for enhancing DDIP.

Molormer is a lightweight self-attention-based method focused on spatial structure of molecular graph for DDIP.

DSN-DDI is a dual-view network that learns drug representations from both single-drug and drug-pair perspectives to capture informative substructures.

SRR-DDI employs a self-attention mechanism to learn robust fine-grained representations of drug substructures.

DAS-DDI dynamically fuses multi-view drug embeddings using an attention mechanism to enhance feature discrimination.

MDF-SA-DDI is a multi-source DDIP approach based on drug fusion, multi-source feature fusion and Transformer self-attention mechanism.

SCATrans is a semantic cross-attention Transformer for DDIP through multimodal biomedical data.

CroMamba-DTA is a feature interaction module to facilitate progressive feature interaction by combining partial cross-attention with the Mamba mechanism.

SmileGNN is a DDIP model by combining SMILES and Graph Neural Network.

Experimental environment of the above methods is shown in Table 2.

**Table 2:** Experimental environment configuration

Component	Parameter
Operating system CPU GPU Memory DL framework	Ubuntu 20.04 LTS Intel Xeon Gold 6226R 2.90 GHz NVIDIA Tesla V100(32 GB) × 4 256 GB DDR4 PyTorch 2.0 + CUDA 11.7

### Dataset

The DDIExtraction2013 corpus (SemEval-2013 Task 9.2) is a widely used benchmark dataset in the field of DDI identification, containing text from MEDLINE abstracts and DrugBank drug labels, totaling 1,025 documents (http://wengzq-lab.cn/ddi/; https://go.drugbank.com/releases) [24, 25]. This dataset labels four types of DDIs (Mechanism, Effect, Advice, Int) and negative cases (no interaction), but its sample distribution is highly unbalanced: there are only 5,002 positive cases, while there are 33,486 negative cases. In the positive example, the number of samples of type "Int" is significantly less than that of type "Effect". This unbalanced distribution is prone to cause the model to favor the majority of classes, affecting the recognition effect on rare types. To alleviate this problem, strategies such as negative example filtering, over-sampling/under-sampling, or adjusting the loss function are often adopted. Its detailed distribution is shown in Table 3.

**Table 3:** The detailed distribution after filtering

DDI type		Training set	Test set
Positive	Advice	824	221
	Effect	1675	359
	Mechanism	1309	301
	Int	188	96
	Total	3996	977
Negative	8987	2049	
Total	12983	3026	

The original text data is from the DDIExtraction2013 dataset, which contains a total of 11,930 drugs and their related DDKs (drug-drug knowledge). In the data preprocessing stage, each sentence and DDK undergo word segmentation, standardization, stop word filtering, punctuation and special symbol removal, and are uniformly converted to lowercase.

### Dataset preprocessing and augmenting

#### Dataset preprocessing

Text Cleaning: All textual data (drug descriptions, social media text) are preprocessed consistently: conversion to lowercase, removal of non-alphanumeric characters and extra whitespace, and filtering of standard English stop words using the NLTK library.

SMILES Standardization: SMILES strings are canonicalized using RDKit to ensure consistent molecular representation. Invalid SMILES sequences (approximately 0.8% of the dataset) are removed from the analysis.

Sequence Length Processing: For computational efficiency, maximum sequence lengths of 250 tokens for SMILES strings and 512 tokens for textual descriptions are established. Longer sequences are truncated, while shorter sequences are padded with zeros.

#### Sample augmenting

To address the severe class imbalance issue in the DDIExtraction2013 dataset, especially for the rare "Int" class (n = 188), during the training period, Synthetic Minority Over-sampling Technique (SMOTE) is specifically used to generate synthetic samples for the severely underrepresented "Int" class. This method creates new instances by interpolating among the existing few class samples in the feature space, effectively extending the training set of key classes while avoiding precise repetition. Oversampling is only applied to the training set. Its sampling strategy is to increase the number of "Int" class samples to match the number of the second rare class, thereby ensuring sufficient representativeness without introducing excessive synthetic data. Moreover, class-weighted cross-entropy loss is employed to assign higher weights to minority classes proportional to their inverse frequency to ensure the optimization process focuses adequately on challenging minority examples.

#### Dataset splitting methodology

Standard Train-Test Split. The original DDIExtraction2013 train-test split contains 12,983 drug pairs (3,996 positive, 8,987 negative) Training samples, 3,026 drug pairs (977 positive, 2,049 negative) test samples. This standard split ensures direct comparability with prior work using this benchmark.

Five-Fold Cross-Validation (5FCV). For robust performance estimation, stratified fivefold cross-validation on the combined dataset (16,009 drug pairs total) is implemented. The stratification preserved the original distribution of DDI types in each fold, addressing class imbalance. In each fold, 4 folds (≈12,807 samples) for training, onefold (≈3,202 samples) for testing.

### Experimental set

Similar to most multimodal DL models [20–22], C2M-Mamba contains multiple trainable hyperparameters, which have a significant impact on model performance. Hyperparameters usually need to be initially set based on experience, and then different parameter combinations are optimized and adjusted through a large number of experiments. The initial hyperparameter Settings of C2M-Mamba are shown in Table 4:

**Table 4:** Initial hyperparameter setting

Hyperparameter	Settings
Input layer dimension Edge feature dimension Dropout rate Hidden layer dimension Deviation parameter range Graph convolutional network layer Jump connection layer Activation function Graph pooling layer	64 64 0.2 64 uniform distribution [-0.2, 0.2] 1 GCN, 2 GAT Two fully-connected layers ReLU Max-pooling

Hyperparameters for feature learning of molecular fingerprint information of drugs are set as, Input layer dimension 64, Hidden layer dimension 64, Dropout 0.2, the dimensions of the first-layer network 512, the dimension of the sub-layer network 256, activation function ReLU. Hyperparameters for one-dimensional sequence feature learning of drugs are set as, dictionary size 66, model dimension 64, the number of attention heads 1, the number of encoding layers 1, the dimension of the feedforward network 128, maximum sequence length 100, Dropout 0.2. The iteration times of network to 3000, Batch-size to 64, dropout rate to 0.5 to avoid over-fitting, and learning rate to 0.001.

The embedding dimensions of biomedical corpus and DDK are two key parameters, which can be adjusted by DDIP experiments on the filtered DDIExtraction2013 dataset. The effect of embedding dimensions on DDIP is verified by experiments of the given train-test distribution dataset. When the embedding dimension of DDK is set as 300, five different embedding dimensions of 50, 100, 200, 300 and 400 are selected. The experimental results show that the optimal configuration with an embedding dimension of 300 is selected, which can maintain a high accuracy rate (75.51%) while controlling training costs.

The experiments are performed on the given train-test distribution and by five-fold cross-validation (5FCV). 5FCV is that, all DDIs are split into 5 subsets, of which four subsets are used as training set to train the model, and the other subset is used as test set for verifying the model, which is repeated 5 times. Average results of precision (*P*), recall (*R*) and *F*1-score (*F*1) are often employed to evaluate the model performance, calculated as follows,

10 $$ \begin{gathered} P = \frac{{1}}{5}\sum\limits_{{l \in S_{DDI} }} {P_{l} } \;,R = \frac{{1}}{5}\sum\limits_{{l \in S_{DDI} }} {R_{l} } ,\;F{1} = \frac{{1}}{5}\frac{{{2}PR}}{P + R} \hfill \\ AUC = \frac{{\sum\nolimits_{i = 1}^{P} {\sum\nolimits_{j = 1}^{N} I } (p_{i} > n_{j} ) + 0.5 \cdot I(p_{i} = n_{j} )}}{P \times N} \hfill \\ \end{gathered} $$

where *S*_DDI_ = {Advice, Mechanism, Effect, Int, Negative} is the DDI label set, *P*_*l*_ and *R*_*l*_ of each instance *l* ∈ *S*_DDI_, *P* and *N* are the numbers of true positive and negative examples, $p_{i}$ and $n_{j}$ are the predicted probabilities of the i-th positive and the j-th negative examples, *I*(.)is an indicator function that returns 1 when the condition is true and 0 otherwise. *P*_*l*_ and *R*_*l*_ are evaluated by,

11 $$ P_{l} = \frac{{\# {\text{ Drug - pair DDI is }}l{\text{ and is classified as }}l}}{{\# {\text{ Classified as }}l}},\;R_{l} = \frac{{\# {\text{ Drug - pair DDI is }}l{\text{ and is classified as }}l}}{{\# {\text{ Drugpair is }}l}} $$

### Experimental results

After concatenating the extracted feature vectors, they are reduced to 150 dimensions through principal component analysis (PCA) and used as the input to the C2M-Mamba model. The drug-drug interaction (DDI) prediction results under the given train-test split are presented in Table 5.

**Table 5:** The DDIP results

Type	Negative	Mechanism	Effect	Advice	Int
Result					
Precision (%)	94.6	77.9	76.5	75.4	55.2
Recall(%)	93.9	73.2	75.6	74.3	61.8
F1(%)	94.2	75.4	76.0	74.8	58.3

As shown in Table 5, the *Negative* class achieves the highest prediction performance, with an F1-score of 94.2%, significantly outperforming other categories. This is primarily attributed to the substantial number of negative instances in the dataset, which dominate the model training and introduce prediction bias. In contrast, the *Int* class yields the lowest results (F1-score = 58.3%), owing to its limited number of instances—accounting for only about 4.7% of positive samples—leading to inadequate model learning. To mitigate the impact of class imbalance, strategies such as negative instance filtering can be employed to improve the recognition performance for rare DDI types.

To evaluate the impact of negative-class samples on model performance, a series of experiments are conducted using a filtered dataset from which all negative instances had been removed. The prediction results under the same train-test split are summarized in Table 6.

**Table 6:** The DDIP results on the dataset without negative-instances

Type	Mechanism	Effect	Advice	Int
Result				
Precision (%)	82.97	78.65	79.14	70.36
Recall(%)	80.13	76.82	78.29	65.88
F1(%)	81.52	77.71	78.71	68.10

By comparing Tables 5 and 6, it is seen that after removing the negative samples, the recognition performance of DDI for all four types of positive samples has been significantly improved. Among them, F1 scores of Mechanisms, Effect, Advice and Int increased by approximately 6.02%, 1.71%, 3.91% and 9.80% points respectively. The results show that the severe category imbalance problem caused by a large number of negative samples in the original dataset seriously affects the model's ability to recognize the DDI types of positive samples. These findings suggest that it is necessary to adopt resampling techniques or design loss functions for class imbalance to improve the prediction effect of DDI.

The experiments are performed by the given train-test distribution and 5FCV, and compared C2M-Mamba with 9 DDIP models: DANN [3], Molormer [8], DSN-DDI [12], SRR-DDI [13], DAS-DDI [14], MDF-SA-DDI [16], SCATrans [18], CroMamba-DTA [22] and SmileGNN [23]. Their hyperparameters, including batch-size, number of hidden units, learning rate and dropout rate, are optimized by 5FCV experiments. Their average results are given in Table 7.

**Table 7:** The DDIP results of 10 DDIP methods

Result	Precision (%)	Recall (%)	F1 (%)	AUC (%)	Training time(hour)
Method					
DANN	74.26	71.23	72.71	80.12	5.2
Molormer	73.58	70.64	72.08	79.83	5.1
SRR-DDI	75.65	72.91	74.25	81.45	7.5
DAS-DDI	77.85	75.10	76.45	83.78	7.1
DSN-DDI	78.92	76.16	77.52	84.91	8.7
MDF-SA-DDI	70.93	69.14	70.02	77.25	12.1
SCATrans	76.11	73.21	74.43	81.26	11.8
CroMamba-DTA	79.44	76.83	78.11	85.42	6.3
SmileGNN	77.18	74.25	75.68	83.17	5.6
C2M-Mamba	82.37	79.65	80.98	88.73	6.5

From Table 7, it is found the proposed C2M-Mamba framework achieves state-of-the-art performance across all evaluation metrics, obtaining the highest scores in Precision (82.37%), Recall (79.65%), F1-score (80.98%), and AUC (88.73%). It significantly outperforms all baseline methods, including the strong performing CroMamba-DTA (F1: 78.11%) and DSN-DDI (F1: 77.52%), while maintaining competitive training efficiency (6.5 h). Notably, our method demonstrates substantial improvements over recent approaches such as MDF-SA-DDI and SCATrans, which require significantly longer training times (12.1 h and 11.8 h respectively) yet yield inferior results. This comprehensive evaluation confirms that C2M-Mamba effectively balances model performance with computational efficiency, establishing it as a robust solution for DDI prediction tasks.

To comprehensively assess the contribution of each component in C2M-Mamba, a lot of ablation experiments under consistent experimental settings are performed, including the same train-test split and 5FCV. The study systematically evaluates the impact of individual modules by selectively removing or replacing them while keeping other factors unchanged. The average precision results for each ablated configuration are reported in Table 8.

**Table 8:** The DDIP precisions of some ablation experiments

Result	Precision (%)
Variant of C2M-Mamba	
Only social media texts Only drug description Only drug SMIELS social media texts and drug SMIELS social media texts and drug description drug description and drug SMIELS Multi-head attention replaces attention pooling No convolutional module No PCA dimensionality reduction No CroMamba module No Max pooling Transformer replaces Mamba Add part-of-speech feature (POS) Complete C2M-Mamba	73.84 65.18 73.35 75.26 74.71 74.49 75.33 72.65 81.64 71.58 71.14 74.65 75.33 82.37

From Table 8, it is indicated that the complete C2M-Mamba model (with precision of 82.37%) is significantly superior to all variants, verifying the effectiveness of its architecture. Specifically: (1) CroMamba has the greatest contribution. After its removal, the precision dropped significantly to 71.58%. (2) SMILES features (73.35%) and convolutional modules (72.65%) are crucial for local structure modeling. (3) In contrast, the selection of embedding methods and PCA dimensionality reduction have a relatively small impact on performance, and part-of-speech features (POS) even have no significant gain. (4) After replacing Mamba with Transformer, the performance declined (74.65%), demonstrating the advantages of Mamba in long sequence modeling. The collaborative work of all components has jointly enhanced the performance of the model.

### Model interpretation and visualization

**Fig. 3:**
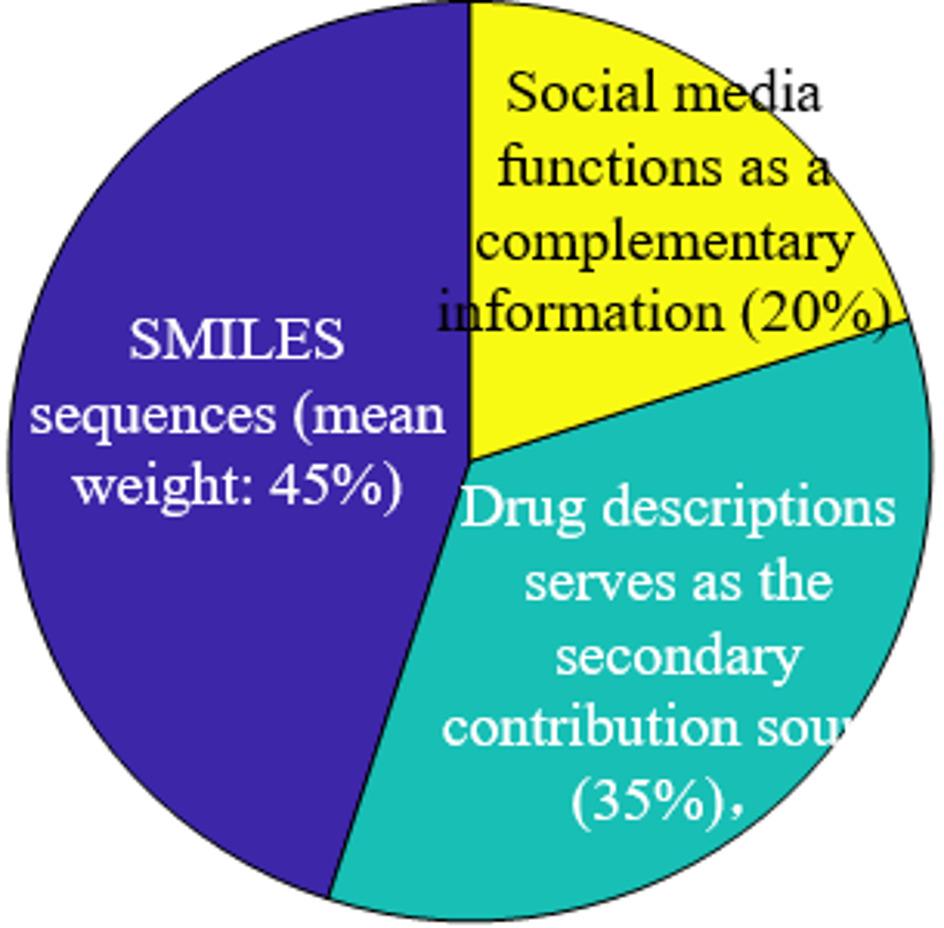
The important of SMILES sequences, drug descriptions and social media tex

To enhance the interpretation of the decision-making process of C2M-Mamba, inspired by Ref [26, 27], visualizations of two critical components are provided. Figure 2 illustrates the partial cross-attention weight distribution across different cross-modal interaction patterns for various DDI samples. Each row represents a drug pair, while each column corresponds to a specific DDI pattern. The deeper the shadow, the higher the attention value, indicating how the cross-modal attention mechanism of C2M-Mamba selectively focuses on the most relevant interaction patterns and captures different semantic relationships between different drug pairs. As illustrated in Fig. 3, the model demonstrates a primary reliance on chemical structural information encoded in SMILES sequences (mean weight: 45%), indicating its recognition of molecular structure as the fundamental basis for DDI prediction. The standardized pharmacological knowledge provided by drug descriptions serves as the secondary contribution source (35%), while social media text functions as a complementary signal (20%), capturing real-world side effects or experiences potentially absent from formal literature. This weighted distribution aligns with the chemical principle that structure determines property, thereby validating the rationality of the model's decision-making process.

### Result analysis

The ablation studies confirm the critical importance of each component in our C2M-Mamba architecture. The complete model achieves superior performance with 82.37% precision and 88.73% AUC, significantly outperforming all variant configurations. Notably, the CroMamba module demonstrates the most substantial contribution—its removal causes the most severe performance degradation, with precision dropping to 71.58% and AUC decreasing to 75.42%. Similarly, the SMILES features (73.35% precision/79.15% AUC) and convolutional modules (72.65% precision/78.83% AUC) prove essential for effective local structure modeling. In contrast, replacing the Mamba component with Transformer architecture results in notably reduced performance (74.65% precision/82.36% AUC), validating Mamba's superiority in long-sequence modeling. The embedding method selection and PCA dimensionality reduction show relatively minor impacts, while part-of-speech features provide negligible benefits. These findings collectively demonstrate that the synergistic integration of all components enables C2M-Mamba to achieve optimal performance in DDI prediction.

The experimental results demonstrate that C2M-Mamba achieves superior performance compared to other models by effectively integrating multiple advanced techniques. The model leverages semantic embedding, combines Mamba and CroMamba to capture both global contextual information and local structural patterns, and employs PCA along with attention pooling to reduce feature redundancy while highlighting the most discriminative information. This multi-component design enables C2M-Mamba to fully exploit complementary strengths across embedding methods, feature extraction mechanisms, and dimensionality reduction strategies, resulting in a robust and high-performing framework for DDIP.

## Conclusion

This paper presents C2M-Mamba, a novel cross-modal framework for drug-drug interaction prediction that effectively integrates molecular structures with textual information. By combining Mamba's efficient sequence modeling with our proposed CroMamba fusion mechanism, the model achieves superior performance (82.37% precision, 80.98% F1-score, 88.73% AUC) while maintaining computational efficiency. Comprehensive experiments and ablation studies validate our architectural choices, demonstrating significant improvements over existing methods. Future work will focus on model simplification and incorporating additional biological data modalities to further enhance prediction capabilities for drug safety applications.

## Data Availability

The dataset used in this manuscript is the DDIExtraction2013 corpus (SemEval-2013 Task 9.2), which is a widely used benchmark dataset in the field of DDI identification. They can be accessed from web links (http://wengzq-lab.cn/ddi/; https://go.drugbank.com/releases) [21, 22].
